# 4-(5-*tert*-Butyl-1,3-dithian-2-yl)-5-chloro-2-phenyl-1,3-oxazole

**DOI:** 10.1107/S1600536808002821

**Published:** 2008-02-06

**Authors:** Yongliang Cui, Lei Liu, Ming Liu, Yousheng Duan, Shangzhong Liu

**Affiliations:** a Department of Applied Chemistry, China Agriculture University, 100094 Beijing, People’s Republic of China

## Abstract

In the title mol­ecule, C_17_H_20_ClNOS_2_, the phenyl and oxazole rings are nearly coplanar with an average deviation of 0.022 Å from the mean plane (*M*). The 1,3-dithiane ring adopts a chair conformation and is twisted in such a way that the C—C_Bu_ fragment lies in *M* (deviations are 0.031 and 0.010 Å, respectively, for the two C atoms).

## Related literature

For details of the pharmacological properties of the GABA [GABA = γ-aminobutyric acid] receptor, see: Wacher *et al.* (1992 ▶). For the related structural series of the GABA receptor, see: Jeffrey (2003 ▶); Naratashi *et al.* (2007 ▶).
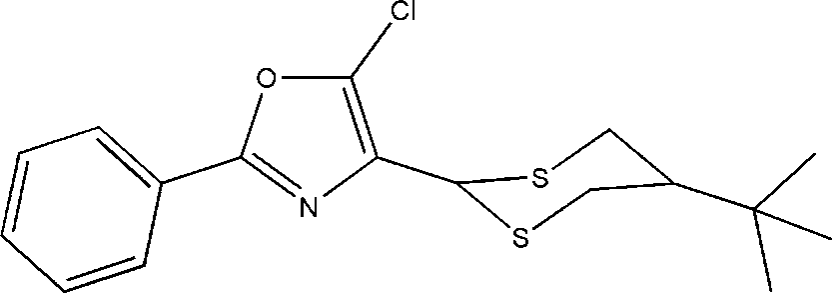


## Experimental

### 

#### Crystal data

C_17_H_20_ClNOS_2_
*M* *_r_* = 353.91
Monoclinic, 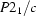

*a* = 7.4543 (15) Å
*b* = 26.222 (5) Å
*c* = 9.4772 (19) Å
β = 104.59 (3)°
*V* = 1792.7 (6) Å^3^
*Z* = 4
Mo *K*α radiation
μ = 0.45 mm^−1^
*T* = 296 (2) K
0.34 × 0.31 × 0.18 mm

#### Data collection

Rigaku R-AXIS RAPID IP area-detector diffractometer
Absorption correction: multi-scan (*ABSCOR*; Higashi, 1995 ▶) *T*_min_ = 0.863, *T*_max_ = 0.924
5683 measured reflections
3135 independent reflections
2788 reflections with *I* > 2σ(*I*)
*R*_int_ = 0.023

#### Refinement

*R*[*F* ^2^ > 2σ(*F* ^2^)] = 0.050
*wR*(*F* ^2^) = 0.130
*S* = 1.10
3135 reflections
199 parameters
59 restraints
H-atom parameters constrained
Δρ_max_ = 0.37 e Å^−3^
Δρ_min_ = −0.23 e Å^−3^

### 

Data collection: *RAPID-AUTO* (Rigaku, 2001 ▶); cell refinement: *RAPID-AUTO*; data reduction: *RAPID-AUTO*; program(s) used to solve structure: *SHELXS97* (Sheldrick, 2008 ▶); program(s) used to refine structure: *SHELXL97* (Sheldrick, 2008 ▶); molecular graphics: *XP* in Siemens *SHELXTL* (Sheldrick, 2008 ▶); software used to prepare material for publication: *SHELXL97*.

## Supplementary Material

Crystal structure: contains datablocks I, global. DOI: 10.1107/S1600536808002821/cv2381sup1.cif

Structure factors: contains datablocks I. DOI: 10.1107/S1600536808002821/cv2381Isup2.hkl

Additional supplementary materials:  crystallographic information; 3D view; checkCIF report

## Figures and Tables

**Fig. 1.**
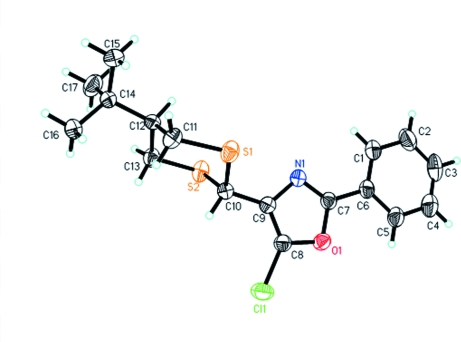
The molecular structure of (I), with atomic labels and 30% probability displacement ellipsoids for non-H atoms.

## supplementary crystallographic information

### Comment

γ-Aminobutyric acid (GABA) receptor of insect exists in their nerve cell and intramuscular cell, and a combinative site of many insecticide and active compounds (Wacher *et al.*, 1992). A large number of related structural series (*e.g.*, trioxabicyclooctanes, thiazines, arylpyrimidines, oxathianes, and dithianes) was synthesized and assayed on GABA receptor to discover novel insecticides (Jeffrey, 2003). Until now, only synthetic compound Fipronil is broadly used to control certain species of insects that have become resistant to most insecticides (Naratashi *et al.*, 2007). In order to further optimize 1,3-dithiane derivative, the title compound, (I), was synthesized. Herewith we present its crystal structure.

In the title molecule, the phenyl and oxazole rings are nearly coplanar with the average deviation of 0.022 Å from the mean plane (*M*). The 1,3-dithiane ring adopts a chair conformation being twisted in such a way, that two-atomic fragment C12—C14 actually lie in *M* with deviations of 0.031 and 0.010 Å, respectively. The crystal structure exhibits no classical hydrogen bonds.

### Experimental

Compound (I) was prepared by the 4 h reaction of 0.8 g (3.85 mmol) of 5-chloro-2-phenyloxazole-4-carbaldehyde and 0.8 g (4.88 mmol) of 2-*tert*-butylpropane-1,3-dithiol in the presence of two drops formic acid used as a catalyst at room temperature with stirring. The resulting mixture was dissolved in chloroform (60 ml), washed with aqueous 10% NaOH (3×20 ml) and H_2_O (3×20 ml), and then dried with anhydrous sodium sulfate. After concentration, the residue was purified by re-crystallization in a mixed solvent of ethyl acetate and petroleum ether. Single crystals suitable for X-ray data collection were obtained by re-crystallization of the crude product from a mixed solvent ethyl acetate and petroleum ether (*v*/*v*, 1/20) as a light yellow crystalline solid (60%), m.p. 453 K.

### Refinement

The H atoms were positioned with idealized geometry (C—H 0.93–0.98 Å), and refined using a riding model with *U*_iso_(H)=1.2 or 1.5*U*_eq_(C).

### Crystal data

**Table:**

C_17_H_20_ClNOS_2_	*F*_000_ = 744
*M**_r_* = 353.91	*D*_x_ = 1.311 Mg m^−^^3^
Monoclinic, *P*2_1_/*c*	Mo *K*α radiation λ = 0.71073 Å
*a* = 7.4543 (15) Å	Cell parameters from 13320 reflections
*b* = 26.222 (5) Å	θ = 2.2–27.5º
*c* = 9.4772 (19) Å	µ = 0.45 mm^−^^1^
β = 104.59 (3)º	*T* = 296 (2) K
*V* = 1792.7 (6) Å^3^	Plate, colourless
*Z* = 4	0.34 × 0.31 × 0.18 mm

### Data collection

**Table:**

Rigaku R-AXIS RAPID IP area-detector diffractometer	3135 independent reflections
Radiation source: rotating anode	2788 reflections with *I* > 2σ(*I*)
Monochromator: graphite	*R*_int_ = 0.023
*T* = 296(2) K	θ_max_ = 25.0º
ω scans at fixed χ = 45°	θ_min_ = 2.7º
Absorption correction: multi-scan(ABSCOR; Higashi, 1995)	*h* = −8→8
*T*_min_ = 0.863, *T*_max_ = 0.924	*k* = −30→31
5683 measured reflections	*l* = −11→11

### Refinement

**Table:**

Refinement on *F*^2^	Secondary atom site location: difference Fourier map
Least-squares matrix: full	Hydrogen site location: inferred from neighbouring sites
*R*[*F*^2^ > 2σ(*F*^2^)] = 0.050	H-atom parameters constrained
*wR*(*F*^2^) = 0.130	*w* = 1/[σ^2^(*F*_o_^2^) + (0.0651*P*)^2^ + 0.55*P*] where *P* = (*F*_o_^2^ + 2*F*_c_^2^)/3
*S* = 1.10	(Δ/σ)_max_ = 0.001
3135 reflections	Δρ_max_ = 0.37 e Å^−^^3^
199 parameters	Δρ_min_ = −0.23 e Å^−^^3^
59 restraints	Extinction correction: none
Primary atom site location: structure-invariant direct methods	

### Special details

**Table:**

Geometry. All e.s.d.'s (except the e.s.d. in the dihedral angle between two l.s. planes) are estimated using the full covariance matrix. The cell e.s.d.'s are taken into account individually in the estimation of e.s.d.'s in distances, angles and torsion angles; correlations between e.s.d.'s in cell parameters are only used when they are defined by crystal symmetry. An approximate (isotropic) treatment of cell e.s.d.'s is used for estimating e.s.d.'s involving l.s. planes.
Refinement. Refinement of *F*^2^ against ALL reflections. The weighted *R*-factor *wR* and goodness of fit *S* are based on *F*^2^, conventional *R*-factors *R* are based on *F*, with *F* set to zero for negative *F*^2^. The threshold expression of *F*^2^ > σ(*F*^2^) is used only for calculating *R*-factors(gt) *etc*. and is not relevant to the choice of reflections for refinement. *R*-factors based on *F*^2^ are statistically about twice as large as those based on *F*, and *R*- factors based on ALL data will be even larger.

### Fractional atomic coordinates and isotropic or equivalent isotropic displacement parameters (Å^2^)

**Table:**

	*x*	*y*	*z*	*U*_iso_*/*U*_eq_	
S1	0.21531 (9)	0.68715 (2)	0.75838 (9)	0.0672 (2)	
S2	0.56576 (9)	0.74615 (2)	0.89160 (8)	0.0656 (2)	
Cl1	0.74022 (11)	0.62257 (3)	0.61190 (8)	0.0840 (3)	
O1	0.7196 (2)	0.57469 (6)	0.84876 (18)	0.0576 (4)	
N1	0.5427 (3)	0.62247 (7)	0.9549 (2)	0.0541 (5)	
C1	0.6072 (4)	0.54919 (11)	1.1975 (3)	0.0732 (7)	
H1B	0.5353	0.5776	1.2042	0.088*	
C2	0.6441 (5)	0.51328 (13)	1.3080 (4)	0.0923 (10)	
H2A	0.5966	0.5174	1.3891	0.111*	
C3	0.7516 (6)	0.47136 (13)	1.2970 (4)	0.0978 (11)	
H3A	0.7789	0.4476	1.3723	0.117*	
C4	0.8183 (5)	0.46438 (12)	1.1775 (4)	0.0869 (9)	
H4A	0.8881	0.4355	1.1703	0.104*	
C5	0.7828 (4)	0.49976 (10)	1.0678 (3)	0.0685 (7)	
H5A	0.8293	0.4950	0.9865	0.082*	
C6	0.6776 (3)	0.54266 (9)	1.0775 (3)	0.0557 (6)	
C7	0.6401 (3)	0.58120 (9)	0.9633 (3)	0.0530 (5)	
C8	0.6628 (3)	0.61672 (10)	0.7651 (3)	0.0567 (6)	
C9	0.5551 (3)	0.64573 (9)	0.8256 (2)	0.0523 (5)	
C10	0.4602 (3)	0.69455 (9)	0.7721 (3)	0.0523 (5)	
H10A	0.4767	0.7014	0.6744	0.063*	
C11	0.1332 (4)	0.75009 (9)	0.6948 (3)	0.0619 (6)	
H11A	0.1585	0.7558	0.6006	0.074*	
H11B	−0.0002	0.7511	0.6810	0.074*	
C12	0.2190 (3)	0.79347 (9)	0.7963 (2)	0.0490 (5)	
H12A	0.2133	0.7835	0.8948	0.059*	
C13	0.4239 (3)	0.79820 (9)	0.7989 (3)	0.0598 (6)	
H13A	0.4711	0.8300	0.8465	0.072*	
H13B	0.4354	0.7999	0.6993	0.072*	
C14	0.1074 (4)	0.84396 (9)	0.7598 (3)	0.0573 (6)	
C15	−0.0878 (4)	0.83563 (13)	0.7799 (4)	0.0839 (9)	
H15A	−0.1582	0.8665	0.7568	0.126*	
H15B	−0.0799	0.8263	0.8793	0.126*	
H15C	−0.1478	0.8088	0.7163	0.126*	
C16	0.0952 (4)	0.86200 (12)	0.6043 (3)	0.0718 (8)	
H16A	0.0248	0.8930	0.5861	0.108*	
H16B	0.0353	0.8363	0.5365	0.108*	
H16C	0.2177	0.8680	0.5927	0.108*	
C17	0.2000 (5)	0.88590 (11)	0.8662 (4)	0.0872 (9)	
H17A	0.1309	0.9170	0.8433	0.131*	
H17B	0.3244	0.8912	0.8580	0.131*	
H17C	0.2026	0.8758	0.9641	0.131*	

### Atomic displacement parameters (Å^2^)

**Table:**

	*U*^11^	*U*^22^	*U*^33^	*U*^12^	*U*^13^	*U*^23^
S1	0.0550 (4)	0.0493 (4)	0.0934 (5)	−0.0030 (3)	0.0113 (3)	−0.0006 (3)
S2	0.0556 (4)	0.0521 (4)	0.0797 (5)	−0.0014 (3)	−0.0003 (3)	0.0031 (3)
Cl1	0.0849 (5)	0.1090 (6)	0.0667 (4)	0.0281 (4)	0.0352 (4)	0.0140 (4)
O1	0.0560 (9)	0.0558 (10)	0.0616 (9)	0.0092 (7)	0.0160 (8)	−0.0001 (8)
N1	0.0581 (11)	0.0478 (11)	0.0585 (11)	0.0033 (9)	0.0184 (9)	0.0039 (9)
C1	0.089 (2)	0.0544 (15)	0.0798 (18)	−0.0037 (14)	0.0279 (15)	0.0077 (13)
C2	0.127 (3)	0.077 (2)	0.078 (2)	−0.0156 (19)	0.0358 (19)	0.0161 (16)
C3	0.120 (3)	0.066 (2)	0.095 (2)	−0.0088 (19)	0.004 (2)	0.0311 (18)
C4	0.089 (2)	0.0623 (18)	0.103 (2)	0.0064 (16)	0.0135 (18)	0.0195 (17)
C5	0.0613 (15)	0.0566 (15)	0.0855 (18)	0.0035 (12)	0.0146 (13)	0.0100 (13)
C6	0.0536 (13)	0.0455 (12)	0.0659 (14)	−0.0071 (10)	0.0112 (11)	0.0033 (11)
C7	0.0498 (12)	0.0492 (13)	0.0595 (13)	−0.0037 (10)	0.0128 (10)	0.0019 (10)
C8	0.0568 (14)	0.0595 (15)	0.0539 (13)	0.0065 (11)	0.0144 (11)	0.0032 (11)
C9	0.0535 (13)	0.0508 (13)	0.0535 (12)	0.0025 (10)	0.0152 (10)	0.0020 (10)
C10	0.0563 (13)	0.0508 (13)	0.0518 (12)	0.0038 (10)	0.0174 (10)	0.0038 (10)
C11	0.0567 (14)	0.0566 (15)	0.0679 (16)	0.0077 (11)	0.0074 (12)	−0.0025 (12)
C12	0.0570 (13)	0.0518 (12)	0.0415 (11)	0.0058 (10)	0.0185 (9)	0.0037 (9)
C13	0.0595 (14)	0.0468 (13)	0.0719 (16)	−0.0006 (11)	0.0145 (12)	0.0041 (12)
C14	0.0698 (15)	0.0535 (14)	0.0535 (13)	0.0113 (11)	0.0246 (11)	0.0070 (10)
C15	0.0820 (19)	0.082 (2)	0.103 (2)	0.0264 (16)	0.0518 (18)	0.0230 (18)
C16	0.0836 (19)	0.0727 (18)	0.0630 (15)	0.0201 (15)	0.0259 (14)	0.0213 (13)
C17	0.119 (3)	0.0591 (17)	0.0819 (19)	0.0193 (17)	0.0230 (18)	−0.0095 (14)

### Geometric parameters (Å, °)

**Table:**

S1—C10	1.808 (3)	C9—C10	1.489 (3)
S1—C11	1.810 (3)	C10—H10A	0.9800
S2—C10	1.812 (2)	C11—C12	1.523 (3)
S2—C13	1.813 (3)	C11—H11A	0.9700
Cl1—C8	1.699 (3)	C11—H11B	0.9700
O1—C8	1.362 (3)	C12—C13	1.526 (3)
O1—C7	1.372 (3)	C12—C14	1.556 (3)
N1—C7	1.294 (3)	C12—H12A	0.9800
N1—C9	1.393 (3)	C13—H13A	0.9700
C1—C6	1.378 (4)	C13—H13B	0.9700
C1—C2	1.383 (4)	C14—C16	1.528 (3)
C1—H1B	0.9300	C14—C15	1.530 (4)
C2—C3	1.380 (5)	C14—C17	1.534 (4)
C2—H2A	0.9300	C15—H15A	0.9600
C3—C4	1.359 (5)	C15—H15B	0.9600
C3—H3A	0.9300	C15—H15C	0.9600
C4—C5	1.369 (4)	C16—H16A	0.9600
C4—H4A	0.9300	C16—H16B	0.9600
C5—C6	1.387 (4)	C16—H16C	0.9600
C5—H5A	0.9300	C17—H17A	0.9600
C6—C7	1.455 (3)	C17—H17B	0.9600
C8—C9	1.335 (3)	C17—H17C	0.9600
			
C10—S1—C11	100.18 (12)	C12—C11—H11B	108.6
C10—S2—C13	98.67 (12)	S1—C11—H11B	108.6
C8—O1—C7	103.14 (18)	H11A—C11—H11B	107.6
C7—N1—C9	105.1 (2)	C11—C12—C13	109.28 (19)
C6—C1—C2	119.7 (3)	C11—C12—C14	112.3 (2)
C6—C1—H1B	120.1	C13—C12—C14	114.3 (2)
C2—C1—H1B	120.1	C11—C12—H12A	106.9
C3—C2—C1	119.6 (3)	C13—C12—H12A	106.9
C3—C2—H2A	120.2	C14—C12—H12A	106.9
C1—C2—H2A	120.2	C12—C13—S2	113.93 (17)
C4—C3—C2	120.8 (3)	C12—C13—H13A	108.8
C4—C3—H3A	119.6	S2—C13—H13A	108.8
C2—C3—H3A	119.6	C12—C13—H13B	108.8
C3—C4—C5	120.0 (3)	S2—C13—H13B	108.8
C3—C4—H4A	120.0	H13A—C13—H13B	107.7
C5—C4—H4A	120.0	C16—C14—C15	109.7 (2)
C4—C5—C6	120.2 (3)	C16—C14—C17	108.7 (2)
C4—C5—H5A	119.9	C15—C14—C17	107.7 (2)
C6—C5—H5A	119.9	C16—C14—C12	112.15 (19)
C1—C6—C5	119.6 (3)	C15—C14—C12	108.8 (2)
C1—C6—C7	119.0 (2)	C17—C14—C12	109.7 (2)
C5—C6—C7	121.3 (2)	C14—C15—H15A	109.5
N1—C7—O1	113.7 (2)	C14—C15—H15B	109.5
N1—C7—C6	128.8 (2)	H15A—C15—H15B	109.5
O1—C7—C6	117.5 (2)	C14—C15—H15C	109.5
C9—C8—O1	110.1 (2)	H15A—C15—H15C	109.5
C9—C8—Cl1	133.3 (2)	H15B—C15—H15C	109.5
O1—C8—Cl1	116.62 (18)	C14—C16—H16A	109.5
C8—C9—N1	108.0 (2)	C14—C16—H16B	109.5
C8—C9—C10	129.0 (2)	H16A—C16—H16B	109.5
N1—C9—C10	123.0 (2)	C14—C16—H16C	109.5
C9—C10—S1	108.47 (16)	H16A—C16—H16C	109.5
C9—C10—S2	109.57 (17)	H16B—C16—H16C	109.5
S1—C10—S2	113.36 (13)	C14—C17—H17A	109.5
C9—C10—H10A	108.4	C14—C17—H17B	109.5
S1—C10—H10A	108.4	H17A—C17—H17B	109.5
S2—C10—H10A	108.4	C14—C17—H17C	109.5
C12—C11—S1	114.70 (17)	H17A—C17—H17C	109.5
C12—C11—H11A	108.6	H17B—C17—H17C	109.5
S1—C11—H11A	108.6		
			
C6—C1—C2—C3	0.3 (5)	C7—N1—C9—C8	−0.8 (3)
C1—C2—C3—C4	−1.5 (6)	C7—N1—C9—C10	179.2 (2)
C2—C3—C4—C5	1.6 (6)	C8—C9—C10—S1	124.2 (3)
C3—C4—C5—C6	−0.5 (5)	N1—C9—C10—S1	−55.9 (3)
C2—C1—C6—C5	0.8 (4)	C8—C9—C10—S2	−111.6 (3)
C2—C1—C6—C7	−179.0 (3)	N1—C9—C10—S2	68.4 (3)
C4—C5—C6—C1	−0.7 (4)	C11—S1—C10—C9	179.42 (16)
C4—C5—C6—C7	179.1 (3)	C11—S1—C10—S2	57.48 (16)
C9—N1—C7—O1	0.4 (3)	C13—S2—C10—C9	179.80 (17)
C9—N1—C7—C6	178.9 (2)	C13—S2—C10—S1	−58.88 (15)
C8—O1—C7—N1	0.2 (3)	C10—S1—C11—C12	−59.4 (2)
C8—O1—C7—C6	−178.5 (2)	S1—C11—C12—C13	67.9 (2)
C1—C6—C7—N1	−1.5 (4)	S1—C11—C12—C14	−164.19 (16)
C5—C6—C7—N1	178.6 (2)	C11—C12—C13—S2	−70.4 (2)
C1—C6—C7—O1	177.0 (2)	C14—C12—C13—S2	162.84 (16)
C5—C6—C7—O1	−2.9 (3)	C10—S2—C13—C12	63.2 (2)
C7—O1—C8—C9	−0.7 (3)	C11—C12—C14—C16	−59.6 (3)
C7—O1—C8—Cl1	178.63 (17)	C13—C12—C14—C16	65.6 (3)
O1—C8—C9—N1	1.0 (3)	C11—C12—C14—C15	62.0 (3)
Cl1—C8—C9—N1	−178.2 (2)	C13—C12—C14—C15	−172.8 (2)
O1—C8—C9—C10	−179.1 (2)	C11—C12—C14—C17	179.6 (2)
Cl1—C8—C9—C10	1.8 (4)	C13—C12—C14—C17	−55.2 (3)
